# Laparoscopic Management of Acute Small Bowel Obstruction in Non-Selected Patients: A 10-Year Experience

**DOI:** 10.3390/jcm11216275

**Published:** 2022-10-25

**Authors:** Nikoletta A. Petrou, Eugenia M. Bonelli, Naomi Watson, Jonathan Wood, Christos Kontovounisios, Nebil Behar

**Affiliations:** 1 Department of General Surgery, Chelsea and Westminster Hospital, London SW10 9NH, UK; 2 Department of Surgery and Cancer, Imperial College London, London SW7 2BX, UK; 3 Department of General Surgery, The Royal Marsden Hospital, London SW3 6JJ, UK

**Keywords:** small bowel obstruction, emergency, laparoscopy, surgical training

## Abstract

The laparoscopic approach to the management of small bowel obstruction (SBO) has been associated with reduced length of hospital stay, complications, and mortality. The laparoscopy-first approach has been limited to highly selective cases to date. In this retrospective observational study, we report our 10-year experience and outcomes within a dedicated Emergency Surgery unit that adopted a non-selective approach in the laparoscopic management of SBO. The surgical approach to all patients that underwent surgery for SBO by an experienced Emergency Surgeon, over a period of 10 years, was divided into two groups of open surgery (OS) or laparoscopy-first (LF). Outcomes included length of stay, complications, mortality, readmission rates and reasons for conversion. Data were reviewed to identify patterns of learning. A total of 189 patients were included in the study. A total of 81.5% were managed with an LF approach. Of these, 25.3% required conversion. LF patients had a similar length of stay, lower 30-day readmission rates and wound complications. Reasons for conversion included need for bowel resection, perforation, and malignancy. Our study had a high intention-to-treat LF population and identified major indications for conversion. As our laparoscopic experience increased, conversion rates substantially reduced. We propose that a LF approach is feasible and can benefit from training within dedicated Emergency Surgery teams.

## 1. Introduction

The use of the laparoscopic approach in planned surgery is well established. However, in emergency surgery, there are obstacles to its uptake. Previous studies on the laparoscopic management of small bowel obstruction (SBO) have been highly selective in terms of patient inclusion [1,2,3,4,5,6,7] and included multiple surgeons and units, with each performing a few cases.

A recent randomised controlled trial comparing laparoscopic to open adhesiolysis for adhesional small bowel obstruction in selected patients noted a reduced post-operative length of hospital stay, reduced complication rates and similar mortality rates to those treated with open surgery [8]. A 2016 systematic review on the same subject also concluded that a laparoscopic approach was advantageous, with results showing reduced morbidity, mortality and surgical infections [9]. More recently, a 2020 systematic review and meta-analysis concluded that the laparoscopic approach is “safe and feasible” and showed no significant difference in severe complications, iatrogenic bowel injury or mortality [10]. However, the laparoscopic management of small bowel obstruction has been associated with considerable conversion rates [5,11].

Volume/outcome studies have been conducted in almost every surgical procedure in the published literature with an observation of better outcomes with a higher number of procedures in individual units. In our unit, emergency general surgery (EGS) is separated at the team level from elective surgery thanks to dedicated emergency surgeons who performed some 75% of the emergency operations within daytime hours, in addition to contributing to the general on-call rota. With a dedicated team of trainees, experience and focused training in EGS develops more rapidly, allowing for the same level of excellence in care that elective surgery enjoys. We are, therefore, able to further extend the indication for the laparoscopic approach to SBO, to a near “Laparoscopy-first” approach level, as will become evident in the data presented here.

In this study, we aimed to examine whether the laparoscopic management of SBO is safe and feasible with potential benefits to patients’ immediate recovery and outcomes following surgery. Our main objectives were to determine the number of patients that were treated with OS and LF, and further determine how many patients in the LF group were subsequently converted to open surgery. Our secondary objectives were to determine the length of stay, complications, 30-day mortality, re-admission rates and reasons for conversion. In addition, we aimed to demonstrate a learning curve.

## 2. Materials and Methods

This was a retrospective observational study. All adult patients that underwent surgery for acute SBO were identified from a prospectively maintained surgical logbook of an experienced Emergency Surgeon in a central London Teaching Hospital, in the United Kingdom. The study spanned from October 2010 to May 2020. Each patient’s electronic record was assessed independently by two reviewers (N.W. and J.W.) and patients who did not meet the criteria for small bowel obstruction were excluded. Therefore, based on their operative records, we excluded patients that were found to have large bowel obstruction, bowel ischaemia not caused by SBO and bowel perforation not caused by SBO). Any uncertainly on patient inclusion was resolved by a third reviewer, either N.P. or N.B. A total of 189 patients with ages ranging from 20 to 90 years were included in the study.

The patients were divided into two main groups of open surgery (OS) and laparoscopy first approach (LF). The intention-to-treat LF group was further divided into three subgroups, based on outcome, as specified in the operative report: patients who were successfully treated with laparoscopy only (LO), patients who underwent a laparoscopically assisted operation (LA, with smaller incision to extract specimen or allow extracorporeal resection and anastomosis) and patients who required conversion to open surgery (CO).

Data were collected from the patients’ electronic records for (1) age, (2) ASA grade, (3) cause of obstruction, (4) post-operative length of stay, (5) 30-day mortality, (6) 30-day readmission, (7) readmission with SBO (up to a year post-surgery), (8) need for re-operation, and (9) complications. We also reviewed the operative records for causes of conversion in the CO group to identify absolute contraindications to laparoscopy. Finally, we examined our year-to-year numbers of OS, LF, LO, LA and CO cases to identify patterns of learning. The study was performed in compliance with the STROBE Statement [12].

## 3. Results

A total of 222 patients were identified from the Consultant Surgeon’s prospectively maintained logbook as having undergone emergency surgery for acute SBO. Upon review of their electronic records, 33 patients did not meet the inclusion criteria of having had surgery for SBO and were excluded from this study. The remaining 189 patients underwent emergency surgery for acute SBO and were included in the study. Of these, 35 patients (18.5%) were treated directly with open surgery (OS) and 154 patients (81.5%) were managed with a laparoscopy-first (LF) approach. Within the LF group, 90 (58.4%) patients were treated with laparoscopy only (LO), 26 (16.9%) patients had laparoscopically assisted (LA) surgery, and 39 (25.3%) were converted to open (CO). The median time to surgery from admission was 1 day for all groups. The patient records for all groups were reviewed up to their first year post-surgery.

### 3.1. Age and ASA-Grade

The mean age was 65 years in the OS group and 61 years in the LF group. This was a truly non-selective study, with the ASA grade not influencing the decision to treat with laparoscopy first. 92.3% of patients with ASA-1 (*n* = 48), 84.1% of patients with ASA-2 (*n* = 53), 67.2% of patients with ASA-3 (*n* = 39), 92.3% of patients with ASA-4 (*n* = 12) and 66.7% of patients with ASA-5 (*n* = 2) were treated with laparoscopy first. Within the LF group, most ASA-1 patients (65.4%) were successfully treated with laparoscopy only and 19.2% required conversion to open surgery. We noted that, as the ASA grade increased, more patients required laparoscopically assisted surgery or conversion to open surgery. For ASA-4 patients, only 23.1% were successfully treated with laparoscopy-only and 46.2% ultimately required conversion.

### 3.2. Causes of SBO

According to the intra-operative findings, the most common causes of obstruction in the OS group were obstruction within a hernia (62.9%, *n* = 22), followed by luminal obstruction, e.g., tumour (25.7%, *n* = 9). In contrast, for the LF group 58.4% (*n* = 90) of cases were due to adhesional obstruction, 16.2% (*n* = 25) were due to obstruction within a hernia and 16.2% (*n* = 25) due to luminal causes. Within the LF group, 68.9% (*n* = 62) of cases of adhesional obstruction were successfully treated with laparoscopy only, 10% (*n* = 9) had laparoscopically assisted surgery and 21.1% (*n* = 19) were converted to open. In contrast, most cases in the LF group with luminal obstruction underwent either laparoscopically assisted surgery (36%, *n* = 9) or conversion to open (40%, *n* = 10).

### 3.3. Length of Stay (LOS) and 30-Day Mortality

The median post-operative LOS for both the OS and LF groups was 7 days. This was lower in the LO group (5 days). The CO group had the highest post-operative median length of stay (9 days). Across all patients, 30-day mortality was 4.8% (*n* = 9). The LF group had a higher mortality of 5.2% (*n* = 8), compared to 2.9% (*n* = 1) in the OS group (OR 1.9). However, within the LF group, patients that were treated with laparoscopy-only had a low mortality of 2.2% (*n* = 2). Mortality within the subgroups was higher (10.3%, *n* = 4)) for patients that were converted to open and those that had laparoscopically assisted surgery (7.7%, *n* = 2). The causes of mortality in these two groups were variable and included pneumonia with respiratory failure, upper GI bleed from perforated duodenal ulcer, neutropenic sepsis, and cardiac failure.

### 3.4. 30-Day Readmission and Recurrence of SBO

Lower 30-day readmission rates were noted in the LF group compared to the OS group (14.9% vs. 20%, OR 0.7). Within the LF group, rates were higher for LO patients (16.7%) and LA patients (15.4%) and lower for CO patients (10.3%). Beyond the post-operative 30-day period, similar rates of readmissions with SBO were noted for the OS and LF groups (11.4% vs. 11.0%, respectively). Furthermore, of those LF patients that were readmitted with SBO, overall, 61.9% required surgery. 50% of OS and CO patients with recurrent SBO required re-operation, compared to 66.7% of LO and LA patients. Therefore, patients who had originally undergone open surgery appeared to have better chances of avoiding re-operation in future episodes of SBO.

### 3.5. Postoperative Complications

The post-operative complications, according to the Clavien Dindo classification, are summarised in Table 1. Minor complications (Clavien Dindo I-II) were present in 42.2% and 37.1% of LF and OS patients, respectively (OR 1.2). Major complications (Clavien Dindo III-IV) were present in 9.1% and 14.3% of LF and OS patients, respectively (OR 0.6). In the OS group, 2.8% of patients had respiratory complications, 8.6% had wound complications and 22.9% required post-operative TPN, compared to the LF group with 11.7%, 7.8% and 18.8%, respectively. Therefore, respiratory complications were more common in the LF group and, particularly, in those patients, who were converted to open surgery (17.9%). Wound infections were lowest in the LO group (1.1%) and need for TPN was similar for patients that had open surgery (22.9%) compared to laparoscopy only (20%).

### 3.6. Need for Resection and Reasons for Conversion

Overall, 42 (22.2%) of the 189 patients required bowel resection. Approximately half the patients (48.5%) in the OS group required resection, in contrast with 16.2% of patients in the LF group. The LF patients that required resection underwent laparoscopically assisted surgery (40%) or were converted to open (60%). Within the CO group, reasons for conversion included the need for resection (43.6%), presence of perforation (10.2%), suspected ischaemia (10.2%), presence of malignancy (10.2%), dense adhesions not suitable to laparoscopic lysis (10.2%), need for better access for diagnostic evaluation (10.2%) and finding of volvulus or malrotation (5.2%).

### 3.7. The Evolution of Choosing an Operative Approach

**Figure 1:**
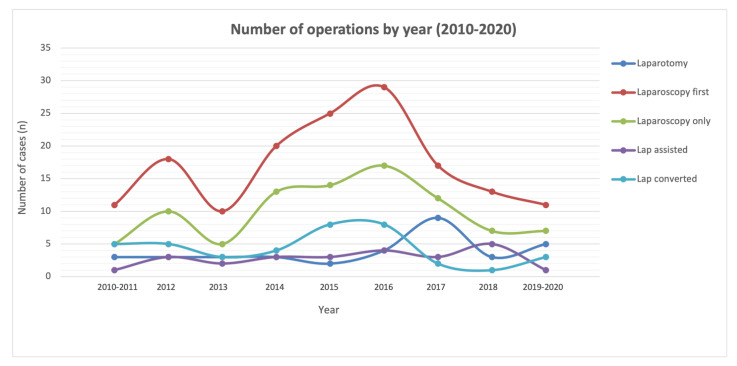
Learning curve of use of laparoscopy for acute SBO (2010–2020).

In the interest of examining our learning curve (Figure 1), we grouped our total operations by year (2010–2013, 2014–2016, and 2017–2020). The results are summarised in Table 2.

We noted that the number of patients undergoing open surgery was initially 18.8%, which then reduced to 10.8%, prior to increasing again to 29.3% in more recent years. Reversely, the number of patients undergoing laparoscopy first was initially 81.2%, which then increased to 89.2%, before more recently reducing to 70.7%. We noted that, in this middle interval of 2014–2016, the number of LF cases that we performed increased, reflecting our keenness to explore the applications of the laparoscopy-first approach. The experience we gained during this middle interval (2014–2016), informed our surgical choices in subsequent years, allowing for us to better identify those patients that are more suited to laparoscopic attempts. This is further reflected in the declining number of patients requiring conversion from 27.1% initially, to 24.1%, to, finally, 10.3% in recent years. We are performing fewer LF cases (70.7%, vs. 81.2% initially) at present, but our conversion rates are also lower (10.3%, vs. 27.1% initially), as our experience has better equipped us to identify patients that are suitable for the laparoscopy-first approach.

## 4. Discussion

### 4.1. Laparoscopy-First as the Preferred Approach and Contraindications for Its Use

According to the Bologna guidelines (2018) for the diagnosis and management of adhesive small bowel obstruction [13], an open surgical approach is generally preferred. A laparoscopic approach is recommended only if the surgeon is sufficiently experienced, the patient has not had more than two laparotomies in the past and the obstruction is due to a single adhesive band [14]. The Bologna guidelines note that there is some modest evidence to suggest that laparoscopy (as opposed to open surgery) results in less extensive post-operative adhesions. The guidelines further recommend that the laparoscopic approach is undertaken only in carefully selected patients, avoiding those with very distended loops or multiple complex adhesions, as the risk of iatrogenic injury is higher in this group of patients.

Our data showed a high intention-to-treat LF population (81.5%), and 75.3% of these had laparoscopy only (LO) or laparoscopically assisted (LA) surgery. In this intention-to-treat LF group, 25.3% required conversion to open surgery. This conversion rate is lower than that noted in other published series [5,15,16]. These data are, overall, in favour of a laparoscopy-first approach. Since pre-operative imaging is not always reliable, the additional diagnostic benefit of this approach helped us to set clear parameters in knowing when to convert.

Our results identified the major reasons for conversion as the need for resection, suspected ischaemia, presence of dense adhesions, perforation, malignancy or malrotation and the need for better access if diagnostic uncertainty remains. We, therefore, propose that the above factors should be considered contraindications to the laparoscopic approach. We also showed that our study was non-selective for patient ASA grade. However, we noted that almost half the ASA-4 patients (46.2%) in the LF group ultimately required conversion to open surgery. We would, therefore, recommend that, for ASA-4 patients, consideration is given to preferential management with direct open surgery.

Adhesional obstruction was predominantly managed with a laparoscopy-first approach, whereas obstruction within a hernia and luminal obstruction were predominantly managed with open surgery. Over two thirds (68.9%) of adhesional SBO cases within the LF group were successfully managed with laparoscopy only. However, LF patients with luminal obstruction predominantly required laparoscopically assisted surgery (36%) or conversion to open surgery (40%) and all LF cases that required resection were either laparoscopically assisted or converted to open surgery. Therefore, we recommend that laparoscopy may serve in an assisting role in cases of luminal obstruction and obstruction within a hernia and has a diagnostic benefit value.

### 4.2. Short and Long-Term Outcomes of the Laparoscopy-First Approach

The LASSO trial (2019) is the only randomised trial comparing the outcomes of open versus laparoscopic adhesiolysis [8]. A total of 100 patients with adhesional SBO were randomised into an open and a laparoscopic group. The authors noted a longer length of post-operative stay in the open group, similar mortality rates and a higher rate of postoperative complications in the open group (although this result was not statistically significant). The study was limited in that it only included patients thought likely to only have a single adhesive band causing SBO.

A systematic review and meta-analysis of 38,927 patients by Quah et al. (2019), comparing open versus laparoscopic adhesiolysis, noted reduced overall mortality and morbidity in the laparoscopic group, including a shorter length of stay. These were retrospective data and note was made that there was likely a selection bias in opting for less complex cases for the laparoscopic approach [17].

There is an overall paucity of studies on the evidence of laparoscopic versus open surgery approach pertaining to acute luminal SBO or SBO with strangulation within a hernia. A 2019 intention-to-treat study by Kohga et al. compared open versus laparoscopic surgery in patients with strangulated small bowel obstruction. They noted that 50% of laparoscopic cases that required bowel resection were ultimately converted to open. In our case cohort, 60% of LF cases that required bowel resection were converted to open and the rsenction in the remaining 40% was laparoscopically assisted [18].

Our data showed a similar median length of stay of 7 days in the OS and LF groups; however, the LO group demonstrated a shorter LOS (5 days), in keeping with the well-established benefits of laparoscopy on post-operative LOS [4,5,7]. Patients that were converted to open had the highest LOS (9 days). Upon review of the individual CO cases regarding the causes for this extended LOS, we noted a higher number of chest sepsis and wound complications in this group, as well as other complications, e.g., anastomotic leak and post-operative bleed, compared to the LO and LA subgroups. These complications cannot be assumed to be directly attributed to our intention-to-treat laparoscopy first approach.

In addition, the lowest 30-day mortality was seen in the LO group (2.2%), compared to 2.9% in the OS group. However, the patients that were converted to open had the highest mortality (at 10.3%) amongst all groups, primarily due to cardiorespiratory causes. Lower 30-day readmission rates were noted in the LF group (14.9%) compared to the OS group (20%), however, we also noted altogether similar readmission rates with recurrent SBO (11.4% vs. 11.0%, respectively). Overall, we did not demonstrate a mortality benefit or a benefit in preventing recurrent SBO with the laparoscopy-first approach.

### 4.3. Strengths and Limitations

This was a retrospective observational study, focusing on the outcomes of an intention-to-treat laparoscopy-first approach. The initial groups of OS and LF were not randomised and, therefore, may not have had similar characteristics at baseline and could be subject to selection bias. Possible confounders include the patients’ comorbidities, the number of previous surgeries a patient had in the past, the presence or absence of organ failure pre-operatively and the overall pre-operative risk as measured by risk prediction scores, e.g., p-possum or NELA scores.

Overall, our study was non-selective for age and ASA grade, which did not influence our decision to proceed with a laparoscopy-first approach. The data were sourced from a prospectively maintained surgical logbook, which reduced recall bias. The main reasons for patients to have OS instead of LF included the known presence of luminal lesions and obstruction within a hernia. Otherwise, most patients (81.5%) underwent a laparoscopic attempt first. Unlike most existing studies in the literature, which focus on adhesional SBO, our study considered all causes of SBO (adhesional, luminal, hernia).

Although we did not observe a mortality or further SBO preventative benefit, 75% of LF cases were completed without requiring conversion to open, and the laparoscopy-only patients had an overall shorter LOS. The additional diagnostic benefit of an LF approach helped guide us to identify indications for early conversion (e.g., need for resection, ischaemia, perforation, dense adhesions, malignancy). Furthermore, our 10-year learning curve showed us that we achieved lower conversions rates in recent years, as we learnt to better identify patients that are more appropriate for the LF approach.

Whilst emergency surgery remains within the competencies of the general surgeon, the added complexity of the laparoscopic approach within the confined space of small bowel obstruction poses challenges to the operation, with pitfalls for the inexperienced. It is, therefore, not surprising that the uptake of this procedure is still low and surgeons prefer early conversion when encountering multiple adhesions, or to avoid laparoscopy altogether when difficult access is suspected based on patient history and radiological findings.

## 5. Conclusions

Large-scale randomised trials are needed to fully evaluate the outcomes of the laparoscopic versus open approach in the management of small bowel obstruction of all causes. We propose that a laparoscopy-first approach to SBO is feasible and, although further research is needed to develop the criteria that would identify patients as suitable candidates for the laparoscopy-first approach, the training and development of skills within dedicated teams to emergency surgery should enable a safe learning curve with equivalent outcomes to selected cases, with all the previously identified benefits.

## Figures and Tables

**Table 1:** 30-day postoperative complications following surgery for SBO.

Clavien Dindo	OS (*n* = 35)	LF (*n* = 154)	LO (*n* = 90)	LA (*n* = 26)	LC (*n* = 39)
I	2	18	11	3	4
II	11	47	22	11	14
III	4	8	5	1	2
IV	1	6	4	-	2
V	1	8	2	2	4

**Table 2:** The evolution of the number of operations performed 2010–2020.

	OS	LF	LO	LA	LC	Total
2010–2013	9 (*18.8%*)	39 (*81.2%*)	20 (*41.6%*)	6 (*12.5%*)	13 (*27.1%*)	48
2014–2016	9 (*10.8%*)	74 (*89.2%*)	44 (*53.0%*)	10 (*12.1%*)	20 (*24.1%*)	83
2017–2020	17 (*29.3%*)	41 (*70.7%*)	26 (*44.8%*)	9 (*15.5%*)	6 (*10.4%*)	58

## Data Availability

Data available upon request from the corresponding author.
